# A Memorial to Dr. Joseph O’Dwyer

**Published:** 1899-05

**Authors:** 


### A MEMORIAL TO DR. JOSEPH O'DWYER.

A COMMITTEE of over forty physicians representing sixteen different medical societies of the city of New York and including representatives of both so-called systems of medicine, has been formed for the purpose of doing honor to the memory of Dr. Joseph O' Dwyer.

The first meeting was held at the New York Academy of Medicine, November 22, 1898, under the chairmanship of Dr. J. D. Bryant and was mainly devoted to organisation. Dr. Geo. F. Shrady was elected permanent chairman, Dr. Alfred Meyer, permanent secretary, and the following committee on scope and plan was appointed: Dr. Dillon Brown, Chairman; and Drs. Robert Abbe, R. G. Freeman, L. Emmet Holt and Louis Fischer. At the second meeting, held at the Academy of Medicine, March 13, 1899, the report of the committee on scope and plan was adopted and now only awaits final action of a meeting of the full committee.

The memorial to Dr. O'Dwyer will probably take an educational form, for by the plan now outlined it is proposed to raise a fund of $30,000, the interest of which shall support two O'Dwyer fellowships in pediatrics, open to competition by physicians who graduate in the United States and to be held by the successful competitors for a period of two years. During this period they must furnish satisfactory proof of their engagement in original research work to a committee of five, one of whom shall be appointed by the president of Harvard University, one by the Dean of the Johns Hopkins Medical School, one by the provost of the University of Pennsylvania, one by the president of the University of Chicago, and one by the president of the New York Academy of Medicine.

Many details of this general plan are still to be arranged, which the secretary will furnish to the medical press of the country so soon as they are finally decided. This preliminary notice has for its object merely to acquaint the profession with the fact that a movement of this nature is on foot, and that an effort will be made to give it the international character so fitting as a memorial to an investigator of international reputation.

